# A TARP Syndrome Phenotype Is Associated with a Novel Splicing Variant in *RBM10*

**DOI:** 10.3390/genes13112154

**Published:** 2022-11-18

**Authors:** Marta Owczarek-Lipska, Fenja Markus, Eva Bültmann, G. Christoph Korenke, John Neidhardt

**Affiliations:** 1 Junior Research Group, Genetics of Childhood Brain Malformations, School of Medicine and Health Sciences, Faculty VI, University of Oldenburg, Ammerländer Heerstr. 114-118, 26129 Oldenburg, Germany; 2 Human Genetics, Faculty of Medicine and Health Sciences, University of Oldenburg, Ammerländer Heerstr. 114-118, 26129 Oldenburg, Germany; 3 Research Center Neurosensory Science, University of Oldenburg, Ammerländer Heerstr. 114-118, 26129 Oldenburg, Germany; 4 Institute of Diagnostic and Interventional Neuroradiology, Hannover Medical School, Carl-Neuberg-Straße 1, 30625 Hannover, Germany; 5 Department of Neuropediatrics, University Children’s Hospital, Klinikum Oldenburg, Rahel-Straus-Straße 10, 26133 Oldenburg, Germany

**Keywords:** TARP, RBM10, Robin’s syndrome, splicing, novel variant, whole exome sequencing, disease-association

## Abstract

TARP syndrome (Talipes equinovarus, Atrial septal defect, Robin sequence, and Persistence of the left superior vena cava) is a rare genetic condition, caused by developmental defects during embryogenesis. The phenotypic spectrum of TARP shows high clinical variability with patients either missing cardinal features or having additional clinical traits. Initially, TARP was considered a lethal syndrome, but patients with milder symptoms were recently described. The TARP-locus was mapped to the gene RNA-binding motif protein 10 (*RBM10*) on the human X-chromosome. We clinically and genetically described a six-year-old boy with a TARP-phenotype. Clinical heterogeneity of symptoms prompted us to sequence the entire exome of this patient. We identified a novel splice variant (NM_005676: c.17+1G>C, p.?) in *RBM10*. A patient-derived cell line was used to verify the pathogenicity of the *RBM10* splice variant by RNA analyses, Western blotting, and immunofluorescence staining. Our molecular genetic findings together with the analyses of progressing clinical symptoms confirmed the diagnosis of TARP. It seems essential to analyze correlations between genotype, phenotype, and molecular/cellular data to better understand *RBM10*-associated pathomechanisms, assist genetic counseling, and support development of therapeutic approaches.

## 1. Introduction

TARP syndrome (TARP) is caused by mutations in *RBM10* (OMIM: 311900). It constitutes a rare congenital syndromic disorder affecting males [1]. Initially, TARP was called “Robin’s syndrome” and it was first described over 50 years ago [2]. Lately, this clinical condition was re-named as TARP and referred to Talipes equinovarus, Atrial septal defect, Robin sequence, and Persistence of the left superior vena cava [3]. Patients affected with TARP typically manifest clubfoot deformity and inborn defect of the heart [1]. Infants with this syndrome often present with multiple problems in feeding and weight gain due to significantly reduced lower jaw, retracted, or displaced tongue, and a high-arched, cleft soft palate. Individuals with TARP also have left-sided superior vena cava [1]. Over time, as more TARP-affected patients were identified by genetic testing, the cardinal clinical hallmarks of the original TARP extended to additional clinical manifestations, including congenital renal, vertebral, and cerebral/cerebellar anomalies, pulmonary hypoplasia, anorectal malformations, aplasia of fingers and toes, and hydronephrosis and hemodynamically significant hypertrophic obstructive cardiomyopathy [4,5]. Atypical clinical presentations were reported in several TARP-cases [4]. Furthermore, mutations in *RBM10* were not only identified in TARP-affected patients but also in patients with lung and pancreatic cancers [3,6,7,8].

Predicting a *RBM10*-associated mutation from the clinical presentation often is challenging due to high clinical variability, sporadic appearance of TARP-affected patients, and the high mortality among TARP-affected boys. The genetic location of the syndrome was firstly mapped to an 11 cm region on human chromosome X (Xp11.23-q13.3), more than 30 years after the first TARP-case was clinically described [1,2]. The initial genetic linkage analyses were performed with only unaffected members of a single 4-generation family, as no living probands with the TARP-phenotype were available at that time [1]. The candidate X-chromosomal region for TARP was then narrowed down to 28 Mb with over 200 genes, including *RBM10* [9]. The identification of a frameshift mutation (NM_005676: c.1893_1894insA) and a nonsense mutation (NM_005676: c.1235G>A) in *RBM10* in two independent families with TARP-affected members finally confirmed the genetic association with TARP [2,9].

*RBM10* generates 5 alternative transcript variants. Depending on the *RBM10*-isoform, the number of coding exons varies between 22 and 24 (Supplementary Table S1). *RBM10* is predominantly involved in processes regulating alternative splicing (AS) of multiple different genes, inhibiting cell proliferation, promoting apoptosis, controlling cell division and replication, and being a tumor suppressor [10,11,12,13]. *RBM10* participates in AS of *Fas* and *Bcl-x* genes promoting exon skipping and/or selecting 5′-splice sites [14]. Moreover, the human *RBM10* may suppress intron splicing thereby regulating exon recognition [13,15]. An in-frame deletion in *RBM10* has previously been associated with TARP [13]. On the molecular level, this mutation caused the loss of the nuclear function of the RBM10 protein, consequently leading to splicing defects in patient-derived lymphoblastoid cells [13]. Mutations affecting either single splicing regulators or essential components of the splicing machinery have been linked to numerous human diseases [13,16,17,18].

According to the literature, various *RBM10*-associated mutations were reported in only 15 unrelated TARP-affected families [19]. The wide spectrum of possible clinical symptoms of *RBM10*-associated mutations put early genetic testing to the forefront of identifying TARP patients.

Herein, we present a six-year-old patient with a novel hemizygous splice site variant in *RBM10* (NM_005676: c.17+1G>C, p.?) identified using a whole-exome sequencing (WES). Our study complements clinical descriptions of previously reported TARP-affected patients, emphasizing the pleiotropic nature of mutations in *RBM10*. Understanding the heterogenous nature of *RBM10*-related diseases is relevant to diagnosis, prognosis, genetic counselling, and therapy development.

## 2. Material and Methods

### 2.1. Probands and Ethical Compliance

Clinical, neurological, and neurophysiological examinations of the TARP-affected patient (IP) were performed in the Children’s Hospitals in Oldenburg (Oldenburg, Germany). Initially, the family was referred for the routine genetic diagnostics and counseling to the Praxis of Human Genetics in Bremen (Bremen, Germany). Molecular genetic analyses and cell culture assays were performed at the Human Genetics Division of the University of Oldenburg (Oldenburg, Germany).

All probands were informed about the course of the study and signed informed written consents before the project started. The study adhered to the tenets of the Declaration of Helsinki and was approved by local ethics committees (Hannover Medical School (MHH) ethics committee, OE9515; Medical Ethic Commission of the University Oldenburg).

### 2.2. DNA Extraction

The genomic DNA (gDNA) was isolated from peripheral blood samples from the IP and his parents, according to the manufacturer’s recommendations (Gentra Puregene Kit, QIAGEN, Hilden, Germany). The gDNA samples were verified for quantity and quality using Qubit^®^ Fluorometer (Thermo Fisher Scientific, Dreieich, Germany).

### 2.3. Whole Exome Sequencing (WES) and Computer Analyses

DNA libraries from the IP were generated using Nextera^®^ Rapid Capture Exome Enrichment Kit (Illumina, San Diego, CA, USA). The WES was performed with NextSeq^TM^ 500 Mid Output Kit at the NextSeq500 platform (Illumina) and paired-end sequencing (2 × 7 bp). The raw WES data were mapped to the human genome reference hg19. Bioinformatic analyses of the WES data, including variant calling and annotations, were made using the Varfeed^TM^/Varvis^TM^ pipeline (VARVIS Version 1.12, Limbus Medical Technologies GmbH, Rostock, Germany). Sequence variants with high/moderate impacts were filtered with allele frequency ≤ 1.5 and sequencing reads quality was verified with the Integrated Genomics Viewer (IGV) [20]. The pedigree and medical history of the family suggested either an autosomal recessive or X-linked mode of inheritance.

### 2.4. Primers, PCR Amplification and Sanger Sequencing

Primers encompassing the first coding exon (exon 2) of *RBM10* (NM_005676; fwd_5′-CGGAGAGCCTTGACAATAAGAG-3′, rev_5′-GCACTATCAGAGCCTAGCAC-3′) were designed [21] and verified for the presence of unwanted single-nucleotide polymorphisms (SNPs) (SNPCheck, Certus Technology, Hems Mews, United Kingdom). PCR amplifications were made with 10 ng of gDNA from the IP and his parents. The PCR conditions, enzymatic purifications of amplicons, and bilateral Sanger sequencing were performed as previously described [22].

### 2.5. Cell Culture of Patient-Derived Fibroblasts

Skin biopsies from the IP and unrelated controls (C1, C2) were used to cultivate patient-derived primary fibroblasts. The biopsies were prepared as previously described [23,24]. Skin fibroblasts were cultured in Minimal Essential Medium (MEM, Biowest, Nuaillé, France) with 20% fetal bovine serum (FBS, Biowest), L-glutamine (Biowest) and antibiotic-antimycotic (Biowest). Cell cultures were growth at 37 °C and 5% CO_2_.

### 2.6. RNA Isolation, cDNA, and Reverse Transcriptase PCR (RT-PCR)

To isolate RNA, fibroblast cell lines from the IP and healthy controls (C1, C2) were pelleted and re-suspended in lysis buffer (Macherey and Nagel, Düren, Germany) with 1% ß-mercaptoethanol (Serva, Heidelberg, Germany). The RNA samples were purified with NucleoSpin^®^ RNA isolation kit (Macherey and Nagel) and the first strand cDNA syntheses were performed with a total of 500 ng RNA, random primers (Metabion, Planegg/Steinkirchen, Germany), and Superscript III Reverse Transcriptase (Invitrogen, Schwerte, Germany). RT-PCR primers (NM_001204468; fwd_5′-TGAGCGTCGACGCTGGTC-3′, rev_5′-CTCCGCACTCTGCTCCTCA-3′) were designed to bind to the 3´ ends of exon 1 and exon 3 of *RBM10*. Primers were used to amplify the cDNA templates using HotFirePol DNA Polymerase (Solis BioDyne) according to standard protocols. The NM_001204468 reference contains a unique nucleotide sequence in the coding part of exon 1, which distinguishes this transcript variant from other *RBM10* transcripts. The amplified PCR products were verified by Sanger sequencing.

### 2.7. Immunocytochemical Staining (ICC)

Skin fibroblasts cell lines from the IP and control (C1, C2) were counted and seeded on coverslips (1.2 × 10^6^ cells per 12 mm coverslip) by overnight incubation in 12-well plates [25]. Afterwards, cells were fixed for 20 min in 4% paraformaldehyde (PFA, Carl Roth, Karlsruhe, Germany) and blocked for 30 min in phosphate-buffered saline (PBS, Chemsolute, Renningen, Germany) with 0.1% Tween 20 (AppliChem, Darmstadt, Germany) and 5% bovine serum albumin (BSA, fraction V, Carl Roth). The ICC staining was performed with primary Anti-RBM10/S1-1 antibody (Abcam, Cambridge, UK, ab72423, 1:300) at room temperature for 2 h. Later, skin fibroblasts were incubated for 2 h at room temperature with secondary antibodies Alexa Fluor 568 (Thermo Fisher Scientific, A-10037, 1:2000). Each incubation was terminated by washing the cells in PBS supplemented with 0.2% Tween 20. Coverslips were mounted with DAPI Fluoromount-G (SouthernBiotech, Birmingham, AL, USA) and slides were analyzed with a fluorescence microscope (Axio Observer, Zeiss, Oberkochen, Germany) and Axiocam 512 mono (4248 × 2832 pixles). Microscope images were examined with ImageJ software (ImageJ, Bethesda, Maryland, USA).

### 2.8. Western Blotting (WB)

Protein pellets were obtained from skin fibroblasts derived from the IP and controls (C1, C2). Preparations of total cell proteins, including cell lysis, protein extractions, and protein quantifications were made as previously described [25]. After that, protein samples (30 µg each) were separated with a 10% polyacrylamide gel and transferred to a polyvinylidene difluoride (PVDF) membrane (pore size: 0.45 µm, Merck Millipore, Burlington, Massachusetts, USA) by applying 45 V for 110 min using a wet blotting system (Mini Trans-Blot Cell, Bio Rad, Hercules, California, USA). Membranes were blocked for 1 h with tris-buffered saline with 0.1% Tween 20 (TBST) and 5% BSA. The overnight incubation with primary antibodies (Anti-RBM10/S1, Abcam ab72423, 1:300 and Anti-GAPDH, Merck Millipore, MAB374, 1:100) was performed at 4 °C. The PVDF-membrane was incubated with horse radish peroxidase (HRP)-linked secondary antibodies (Novus Biological, NBP2-30348H and NB7539, both 1:5000) at room temperature for 1 h and with enhanced chemiluminescence solution (ECL, Thermo Fisher Scientific). All antibodies were initially diluted in TBST with 5% BSA. The WB-signals of RBM10 proteins were visualized with ChemiDoc MP Imaging System (Bio Rad).

## 3. Results

### 3.1. Clinical Data and Initial Routine Genetic Diagnostics

**Figure 1:**
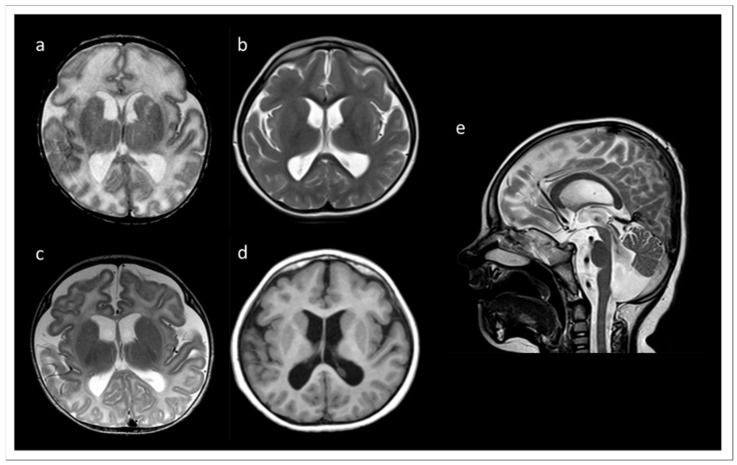
MRI records of the TARP-affected patient. (**a**) At term equivalent age the cerebrum was swollen with increased T2 signal of the supratentorial white matter and a simplified gyral pattern, especially frontal. In the further course, microcephaly with simplified gyral pattern and myelination delay were visible. (**b**) T2 at the age of 3 months. (**c**) T2 at the age of 3.5 years. (**d**) T1 at the age of 3.5 years. (**e**) T2 at 3.5 years of age showing concomitant ventriculomegaly, thickening of the corpus callosum, and pontine and vermis hypoplasia.

We report a six years old boy with a developmental disorder phenotype, born to non-consanguineous and healthy Lithuanian parents. No further family members were reported to be affected with similar symptoms. The IP was delivered by Caesarean section at 33 weeks and 2 days of gestation, because a pathological cardiotocographic (CTG) picture and dystrophy was detected. His birth weight was 1410 g (z-score −1.66), body length 42 cm (z-score −1.06), and head circumference 29 cm (z-score −1.45). The newborn patient was hospitalized over 9 weeks because of a profound drinking disorder with failure to thrive requiring tube feeding. In the first MRI at term equivalent age the cerebrum appeared swollen due to an immature sulcation with less numerous and broad gyri with shallow sulci, especially frontal. The T2 signal of the supratentorial white matter was significantly increased (Figure 1a). During follow up at the age of 3 months, the process of gyration and sulcation was completed. The brain was microcephalic with a simplified gyral pattern. Signs of myelination were slowly increasing, but still not age-appropriate (Figure 1b). At the age of 3.5 years, myelination was clearly advanced, but not yet complete (Figure 1c,d). There was concomitant ventriculomegaly, thickening of the corpus callosum, and pontine and vermis hypoplasia (Figure 1e).

Profound myoclonus was observed; however, the EEG diagnostic did not show any remarkable epileptic findings. Generally, the IP manifested signs of a combined developmental delay. Expanded renal pelvic calyx system on both sides with relatively little medulla-cortex differentiation and gastroesophageal reflux were also observed. An abdominal sonography was performed due to undescended left testicle, which was then operationally corrected. A congenital bronchopulmonary dysplasia significantly improved over time, so that the IP needed oxygen supplementation only during sleep. The IP made slow developmental progress in all areas, e.g., at the age of about 30 months he started to shorty stand up, tried to crawl, began to make voices, and began to react and recognize simple words of his parents. The head circumference at the age of 18 months was under the 3rd percentile. However, the cause of the developmental disorder in the patient remained unclear. TARP was not originally considered for the main diagnosis, especially due to poorly recognizable features of this syndrome in the IP and a lack of several characteristic traits, such as clubfeet and cardiovascular defects. Over time, the re-verification of clinical symptoms in the IP revealed several clinical similarities to other TARP-affected patients, indicating a high probability of TARP in the IP. Despite the fact that some of the cardinal traits of TARP were initially unrecognizable in the IP, the patient showed evidence of the Robin sequence and microcephaly later. The MRI recordings documented simplified gyration, especially of the frontal lobes, which has also been reported in other cases diagnosed with TARP. Typical for children with TARP is a respiratory disorder with oxygen dependence, which was also found in the IP. The cleft palate and renal abnormalities described in the IP were previously observed in other TARP-affected patients.

### 3.2. Routine Cytogenetics and Molecular Gene Testing

Routine pre- and postnatal diagnostic testing was performed on the IP and the obtained results were verified with the parental material.

Amniocentesis was carried out due to bilateral plexus cysts detected during ultrasound examinations of the fetus. These prenatal cytogenetic analyses revealed a Robertsonian translocation between chromosomes 13 and 14 (45,XY,t(13:14)(q10;q10), which was with high probability predicted to not be phenotypically relevant, as the same chromosomal aberration was also found in the healthy mother. A postnatal array-CGH genetic testing additionally showed a heterozygous deletion in chromosome 7p22.1 (arr[hg19]7p22.1/4,862,228–6,000,562)x1) and a heterozygous duplication in chromosome 7q31.2 (arr[hg19]7q31.2/116,571,043–117,135,007)x3) in the affected child as well as in his healthy father, suggesting non-pathogenic findings. A routine molecular gene diagnostic using multiplex ligation-dependent probe amplification (MLPA) for genes involved in brain development (*PAFAH1B1*, *DCX*, *ARX*, *TUBA1A*, *RELN*, *POMT1*, *POMGNT1*, *FKRP*, *FKTN*, and *POMT2*), but again provided no evidence of variants that could explained the phenotype of the unusual cerebral malformation with simplified gyration in the IP.

### 3.3. WES Outcome

WES was performed in the IP. Three DNA libraires, originating from the IP and two different individuals, who were not related to the study, were multiplexed for next generation sequencing (NGS) of the entire coding exons. The 3-plex sequencing run resulted in 28 Gb of the raw WES data, showing Q30-score of 93.4% cluster density of 226 K/mm^2^ and cluster passing filter of 92%. De-multiplexing and variant calling uncover 146.70 M sequencing reads and 88.85% of 20× targeted coverage and 81,103 sequence variants (including 30,631 homozygous and 43,069 heterozygous variants) in the sequencing data of the IP.

To verify sequence variants in genes associated, related, and/or predicted to cause various brain malformation syndromes, the WES data set was initially analyzed with an in-house panel of 500 genes. The panel was assembled on the bases of searching medical databases for clinical features overlapping with those described in the IP. This resulted in an extended list of plausible different diagnoses. Notably, TARP was not included in this panel, because of little indicative phenotypic traits for this syndrome in the IP. From 33,000 sequencing variants detected in the patient, only 4 variants (*CYFIP2*, *KIF11*, *PHC1*, and *SYNE1*) passed the initial filtering criteria regarding reads quality, molecular consequences, and rarity. However, these sequence variants did not fulfill requirements of significant overlap with the most relevant clinical presentations of the IP. Performing the analyses of all sequence variants, irrespective of the gene panel, but retaining previous filtering criteria, we found 896 heterozygous, 79 homozygous, and 4 hemizygous sequence variants. All sequence variants, except one sequence variant in *RBM10*, were excluded from further analyses because they did not meet essential criteria for being a candidate sequence variant for the disease. In summary, research analyses of the WES data identified a likely pathogenic and novel hemizygous splice site sequence variant (NM_005676: c.17+1G>C, p.?) in *RBM10* in the IP.

The sequence variant in *RBM10* was the only promising candidate identified from WES. Therefore, our molecular genetic diagnosis suggested the diagnosis of TARP in the affected boy. Reassessment of the clinical symptoms that manifested during development of the IP finally confirmed the genetic assumption of the *RBM10*-associated TARP.

### 3.4. Familiar Genotyping Analyses

**Figure 2:**
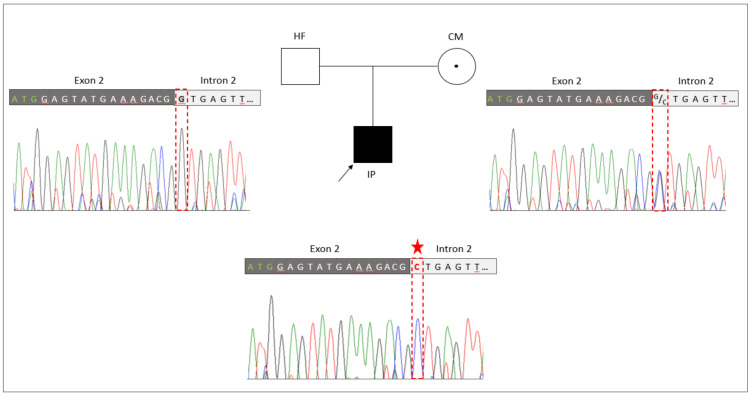
Genetic identification of the novel splice variant (NM_005676: c.17+1G>C, p.?) in *RBM10*. The pedigree of the family with the TARP-affected child (IP), the healthy father (HF) and the healthy carrier mother (CM). Familiar genotyping analyses of the *RBM10*-associated splice variant (NM_005676: c.17+1G>C, p.?) confirmed that the splice variant was inherited by the IP from the heterozygous CM, while the HF presented the normal reference allele. The sequencing peaks showing the nucleotide position of the splice variant is highlighted by a red frame and the mutated allele is indicated by an asterisk.

The novel splice site sequence variant in *RBM10* (NM_005676: c.17+1G>C, p.?) was verified by Sanger sequencing in the TARP-affected boy and his healthy parents (Figure 2). The co-segregation analyses showed that the hemizygous variant (NM_005676: c.17+1G>C, p.?) was inherited from the mother to the IP. The mother was a heterozygous carrier of the mutation. The father of the patient showed a hemizygous reference allele, as expected. A second independent genotyping analysis with different DNA aliquots from the probands confirmed the co-segregation. The maternally inherited hemizygous *RBM10* splice site variant (NM_005676: c.17+1G>C, p.?) in the IP was consistent with the X-linked recessive mode of inheritance associated with *RBM10* mutations.

### 3.5. Transcript and Protein Analyses (RT-PCR, ICC, and WB)

**Figure 3:**
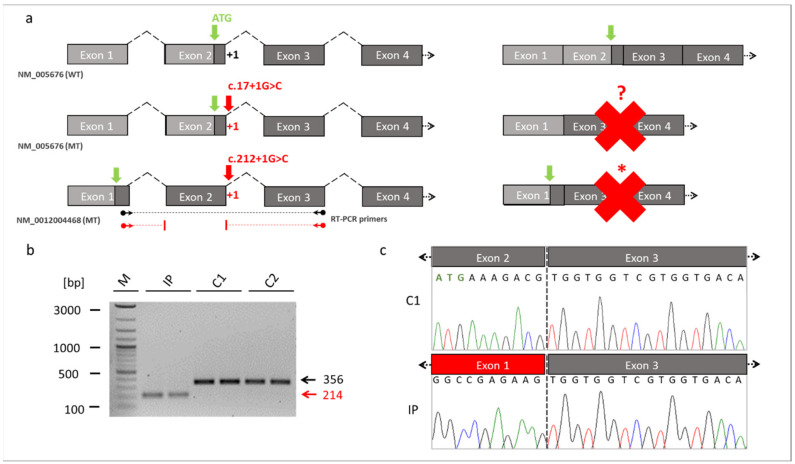
Skipping of the exon 2 in *RBM10*. (**a**) Schematic drawings of the exons of *RBM10* based on the reference transcript variant 1 (NM_005676) and transcript variant 5 (NM_0012004468). The location of the novel splice variant at the junction site between exon 2 and intron 2 is shown in red. The identified splice variant at position +1 of the donor splice site (red arrow) resulted in exon 2 skipping and loss of the start codon (green arrow) in the *RBM10* transcript variant 1. It unclear whether an alternative start codon was generated (red question mark). The same splice variant caused a frameshift and abnormal stop codon (red asterisk) in the *RBM10* transcript variant 5. The dark grey boxes referred to the coding exons, while the light box referred to the noncoding exons. Binding sites of the RT-PCR primers are shown by the black (WT) and red (MT) arrows ending in spheres (transcript NM_0012004468). (**b**) RT-PCR from patient-derived fibroblast cell lines. The RT-PCR showed a reduced fragment size in the TARP-affected patient (reduced by 142bp, NM_0012004468: c.212+1G>C, p.Glu24Valfs127*). The reduced intensity of the IP´s fragment indicated that NMD mechanisms may have partially degraded *RBM10* transcripts. (**c**) Sanger sequencing of the RT-PCR products confirmed skipping of the exon 2 in the IP. The missing sequence in the TARP-affected patient corresponded to exon 2 of the *RBM10*. Abbreviations: IP (TARP-affected patient), C1 and C2 (control individuals), WT (reference), MT (mutant), NMD (nonsense-mediated decay) M (1 kb Plus DNA ladder, New England Biolabs), bp (base pairs).

**Figure 4:**
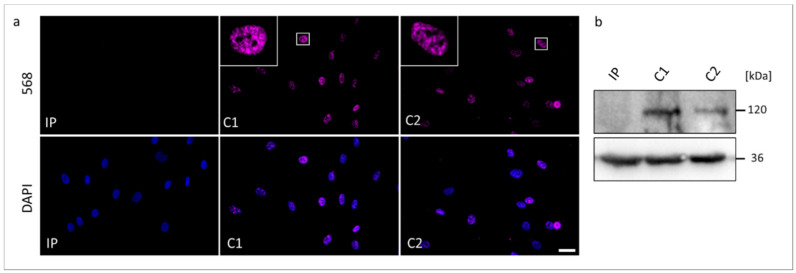
The RBM10 protein analyses in skin fibroblasts from the TARP-affected patient (IP) and unrelated control cell lines (C1 and C2). (**a**) Immunocytochemical detection of the RBM10 was performed with antibodies against RBM10 (magenta). Nuclear DNA was stained by DAPI (blue). The scale bar corresponds to 30 μm. (**b**) Western blotting showed the expected molecular weight of RBM10 of 120 kDa in the protein samples from C1 and C2, while RBM10 was not detectable in the protein sample from IP. GAPDH (36 kDa) served as a loading control.

To further verify pathogenic molecular processes caused by the novel identified *RMB10* splice variant we performed RNA (Figure 3) and protein analyses (Figure 4).

*RBM10* is known to be expressed by 5 transcript variants (Supplementary Table S1). We referred the identified novel splice variant either to the commonly used *RBM10* transcript variant 1 (NM_005676) or to the longest *RBM10* transcript variant 5 (NM_001204468). The novel splice variant (NM_005676: c.17+1G>C; NM_001204468: c.212+1G>C) affected the splice donor site of exon 2 at the first position of intron 2. It was thus likely that this variant interfered with splicing of the second exon of *RBM10* (Figure 3a). We cultured a patient-derived cell line from which we analyzed the possible splice defect in *RMB10*. RT-PCR analyses revealed a shorter amplification product in the IP compared to controls (Figure 3b). This finding suggested a splice defect in the IP. Sanger sequencing of the RT-PCR amplicons confirmed the splice defect and found that exon 2 (coding for RNA recognition motif (RRM) and responsible for RNA binding) of the IP was skipped during splicing. Thus, the new splice variant in *RBM10* resulted in the exon 2 exclusion in the patient described herein (Figure 3c).

We performed Western blotting and ICC to compare possible changes in the expression and cellular localization of the RBM10 protein using the IP-derived fibroblasts (Figure 4). In ICC analyses, both controls C1 and C2 showed overlapping signals with the DAPI gDNA staining, underlining the nuclear localization of the normal RBM10 protein. Significantly, fibroblasts from the TARP-affected patient did not show detectable signals of RBM10 protein in the nucleus (Figure 4a). Therefore, the ICC staining suggested a strongly reduced or degraded RBM10 protein in the IP as a consequence of the mutation-induced splice defect. The ICC results (*n* = 3) were confirmed by WB analyses (*n* = 3). WB of whole-cell protein lysates detected the RBM10 protein at the expected size of 120 kDa in controls (C1 and C2) but failed to find RBM10 signals in the TARP-affected patient (Figure 4b). Data presented in Figure 3b further suggested that the novel *RBM10* splice variant (NM_0012004468: c.212+1G>C, p.Glu24Valfs127*) may lead to nonsense-mediated mRNA decay (NMD) of the patient´s transcripts. It therefore is plausible that NMD contributed to the lack of RBM10 signals in ICC and WB (Figure 4).

In summary, our results indicated that the novel *RBM10* splice donor site variant (NM_005676: c.17+1G>C, p.?) caused exon 2 skipping and loss of function of the RBM10 protein in the TARP-affected patient.

## 4. Discussion

**Figure 5:**
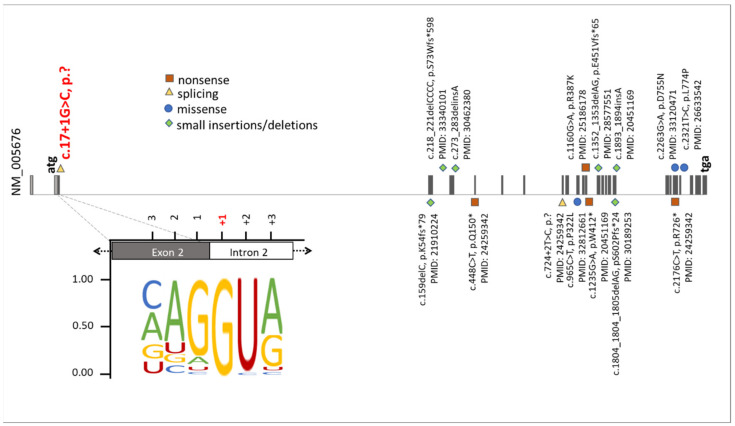
Summary of known mutations in the *RBM10* gene. The presentation of *RBM10* mutations was prepared on the bases of data from the HGMD professional, release 4955. The green diamonds, orange squares, blue circles, and yellow triangles correspond to small insertions/deletions, nonsense mutations, missense mutations, and splice variants, respectively. Additionally, the position of the novel splice variant described herein (NM_005676: c.17+1G>C, p.?) was highlighted on red. The representation of the canonical donor splice sites [26] showed the highest nucleotide conservation at +1. This emphasizes that an exchange of the +1 nucleotide is likely to cause mis-splicing of *RBM10* transcripts.

Originally, four cardinal clinical symptoms (Talipes equinovarus, atrial septal defect, Robin sequence, and persistence of the left superior vena cava) were defined to be sufficient to diagnose TARP syndrome in a patient [2]. Nowadays, it is documented that the variety in the degree and occurrence of TARP-associated symptoms is much broader and more complex than previously thought [19]. The availability of modern molecular genetic technologies (e.g., NGS, WES), as well as the possibility of using them in genetic diagnostics, enabled the identification of additional patients with the rare *RBM10*-mutations associated with TARP in recent years. It was possible to enlarge the phenotypic spectrum of this genetic condition. Due to a highly heterogenous nature of TARP and a pleiotropic character of *RBM10* mutations, together with a small total number of reported TARP-affected cases, our understanding of phenotype-genotype correlations is still incomplete (Figure 5). The phenotypic variability of TARP has important consequences on morbidity and mortality of patients. The first described TARP-affected individuals died in the first days or months of life [2]. Consequently, TARP was considered a syndrome with early lethality. The identification of additional *RBM10* mutations documented patients with milder *TARP* symptoms surviving several years [3,7,25]. Thus, it is essential to further search for new TARP-affected patients to better understand pathomechanisms of the disease and to assist genetic counseling in the diagnosis and prognosis of this genetic syndrome. We speculate that genetic modifiers as well as genes being under expression control of *RBM10* may also influence the expressivity of TARP symptoms.

Herein, we clinically and genetically described a male patient with a TARP phenotype. In the infant patient, it was challenging to determine the TARP diagnosis due to poorly defined main features of this syndrome. The use of WES (analysis of the entire exome of the IP) enabled the early genetic diagnosis and the identification of a novel splice variant (NM_005676: c.17+1G>C, p.?) in the *RBM10* gene (Figure 2). Following the genetic diagnosis, further development of the patient’s clinical symptoms was in line with the TARP diagnosis. Our data suggest that infant patients with mild, unclear, or TARP-like symptoms are transferred to WES or genome-wide genetic analyses—a prerequisite for continuous and targeted monitoring of the patients’ development as well as the treatment of live-threatening genetic diseases.

It is important to note, that of the majority of reported TARP-affected patients died before the age of 18 months [2,3,4,19]. Recently, it has been suggested that the expression of specific *RBM10* transcripts in combination with the location of mutations in relevant functional protein domains may influence the severity and lethality of TARP-affected individuals [19]. Indeed, the majority of those TARP-affected patients with early mortality were affected by severe *RBM10* mutations, i.e., pathogenic, or likely-pathogenic mutations including frameshift or nonsense mutations [19]. However, patients with frameshift mutations have also been described to survive several years, where the oldest reported TARP-affected individual was over 20 years old [7,27]. As frameshift and nonsense *RBM10*-mutations were frequently reported, missense and splice variants were only described occasionally. Recently, a mild form of TARP with developmental delay and dysmorphic traits was reported in the patient having a missense mutation (NM_005676: c.965C>T, p.P322L) in the RRM2 RNA binding domain in *RBM10* [28]. It has been speculated that the biological consequence of this missense mutation and its location in the gene may lead to a milder form of the disease [19]. Only a single splice variant in the *RBM10* gene related to TARP was reported so far. The *RBM10* splice mutation (NM_005676: c.724+2T>C, p.?), which was predicted to be deleterious and was found de novo in a simplex case [3]. Comparably to the case described herein, the child was clinically not diagnosed with TARP during infancy, as it showed only one of four cardinal features. The patient was alive at the time of publication and was about 20 months old [3].

We identified a novel splice variant (NM_005676: c.17+1G>C, p.?) in *RBM10* associated with TARP symptoms. Mutations in canonical splice sites (acceptor and donor) strongly affect evolutionary conserved exon-intron boundaries. These variants lead to serious biological consequences that alter the interaction between pre-mRNA and splicing factors [29]. Consequently, defective removal of exons or introns may lead to changes in the open reading frame and/or coding sequences of transcripts. We showed that the novel splice variant (NM_005676: c.17+1G>C, p.?), changing the highly conserved first canonical nucleotide in the donor splice site, interfered with normal splicing of *RBM10* pre-mRNAs (Figure 5). Mutations at the splice donor site frequently disrupt proper binding of the splicing factor U1snRNP with the pre-mRNA, a molecular mechanism which leads to splice defects and is associated with different diseases and syndromes [30,31]. In the patient described herein, the novel splice variant removed the initiation codon of exon 2 in transcript variant 1 (NM_005676: c.17+1G>C, p.?), whereas skipping of exon 2 resulted in a frameshift and early stop codon in transcript variant 5 (NM_0012004468: c.212+1G>C, p.Glu24Valfs*127) (Figure 3). It is worth mentioning that an incorrect exon recognition due to splicing mutations may also lead to intron retention (IR) [32,33]. IRs that are induced by splice site mutations frequently cause pathogenic alterations of genes expression [30,33,34,35,36]. As intron 2 contains 21816 nucleotides, a complete IR is unlikely to be detected by standard RT-PCR applications. Although we did not detect transcripts including intron 2 sequences (Figure 3b,c), we cannot rule out the possibility of complete or partial IR contributing to the splice defects in the IP. Notably, naturally occurring IR has been associated with expression regulation of genes involved in different biological processes, e.g., cellular differentiation [34], cellular aging [35], and cellular response [36].

It is unclear whether the TARP-phenotype in the patient described herein is caused and/or modulated by splicing alterations of transcripts that are regulated by functional *RMB10*. Interestingly, mutations in several genes controlled by *RBM10* can result in clinical symptoms that mirror those seen in TARP [13]. This includes severe brain malformations, mental retardation, seizures, psychomotor developmental delay, and cardiac, urinary, and gastrointestinal abnormalities (the associated genes are: *DNML* [37], *CEP290* [38], *CASK* [39], *PIGN* [40]). Furthermore, mutations in the genes *ECEL1* [41] and *LHB* [42], are associated with skeletal muscle and limb anomalies as well as hypogonadism [13]. Despite the fact that we did not identify any sequence changes in coding regions of these particular genes, we cannot exclude the possibility of sequence changes in their non-coding and/or regulatory regions. It is tempting to speculate that the mild TARP-phenotype observed in the patient described herein might be modulated by expression or splicing differences in the genes regulated by *RBM10*. This assumption requires further elucidation.

Our analyses showed that the *RBM10* protein product was not detectable in the IP´s cell line, suggesting that the mutation led to molecular consequences on the protein production, stability, or maintenance. NMD mechanisms may also have contributed to reduced RBM10 transcript and protein concentrations in the IP-derived cell line (Figure 3b and Figure 4). Previously reported *RBM10* mutations did not show obvious preferences towards specific types or positions of mutations. They seem to be scattered throughout the gene (Figure 5). Therefore, the genotype-phenotype correlations between the known *RBM10*-associated mutations and TARP, as well as the prediction of severity and survivability in TARP-affected patients still remain inconclusive.

In summary, our molecular genetic findings support that the mutation drastically impacts the function of the protein. Nevertheless, the IP described herein manifested TARP with a survival over 6 years. Our data lead to the speculation that modifying factors exist that contributed to the milder TARP-phenotype in this patient. Identifying such modifiers would also raise the chance for targeted treatments of *RBM10*-associated patients. Our study further shows that early genetic diagnostics can support clinical diagnosis, especially in patients with complex syndromic symptoms. A better understanding of the correlation between molecular, genetic, and clinical findings will be essential to treat patients and to development novel therapeutic approaches.

## Data Availability

The data presented in this study are available on request from the corresponding author. The data are not publicly available due to ethical restrictions.

## Supplementary Materials

The following supporting information can be downloaded at: https://www.mdpi.com/article/10.3390/genes13112154/s1, Table S1: Transcript variants of RBM10 and the nucleotide position of the novel splice variant.
