# Supplementary material for: A TARP Syndrome Phenotype Is Associated with a Novel Splicing Variant in RBM10
Source: Genes (Basel). 2022 Nov 18;13(11):2154. doi: 10.3390/genes13112154 (PMC9691016; doi:10.3390/genes13112154)
Supplement: Supplementary file 1 [file genes-13-02154-s001.zip › genes-1953148-supplementary.pdf]

Supplementary material

Supplementary Table S1. Transcript variants of *RBM10* and the nucleotide position of the novel splice variant.

GenBank number	Number of exons (coding)	Transcript length (bp)	CDS	Start codon	Stop codon	Position of the splice site variant
NM_152856 (tv2)	23 (22)	2559	391-2949	exon 2	exon 23	c.17+1G>C
NM_001204466 (tv3)	23 (22)	2562	391-2952	exon 2	exon 23	c.17+1G>C
NM_001204467 (tv4)	24 (23)	2790	391-3180	exon 2	exon 24	c.17+1G>C
NM_005676 (tv1)*	24 (23)	2793	391-3183	exon 2	exon 24	c.17+1G>C
NM_001204468 (tv5)	24 (24)	2899	227-3214	exon 1	exon 24	c.212+1G>C

* commonly used reference sequence for *RBM10*; tv – transcript variant.
